# Giant and Calcified Post-Infarction True Left Ventricular Aneurysm: What to Do?

**DOI:** 10.5935/abc.20150087

**Published:** 2016-03

**Authors:** Alexandre Hideo Kajita, Marcos Danillo Peixoto Oliveira, Fernando Reis Menezes, Marcelo Franken, Luciano Moreira Baraciolli, José Carlos Nicolau

**Affiliations:** Instituto do Coração - Universidade de São Paulo, São Paulo, SP - Brazil

**Keywords:** Myocardial Infarction / complications, Pulmonary Disease, Chronic Obstructive, Heart Failure, Heart Aneurysm, Diagnostic Imaging

## Introduction

A calcified true left ventricular aneurysm (LVA) is a serious complication in the chronic phase post an acute myocardial infarction (MI); however, it is seldom observed in modern clinical practice¹. LVA may be asymptomatic; however, it can be the cause of refractory heart failure, sustained ventricular arrhythmias, and arterial embolism. The indications for left calcified ventricular aneurysmectomy remain controversial.^1-4^ We report a case of a giant, true LVA that was reasonably managed clinically in an inoperable patient.

## Case Report

**Figure 1:**
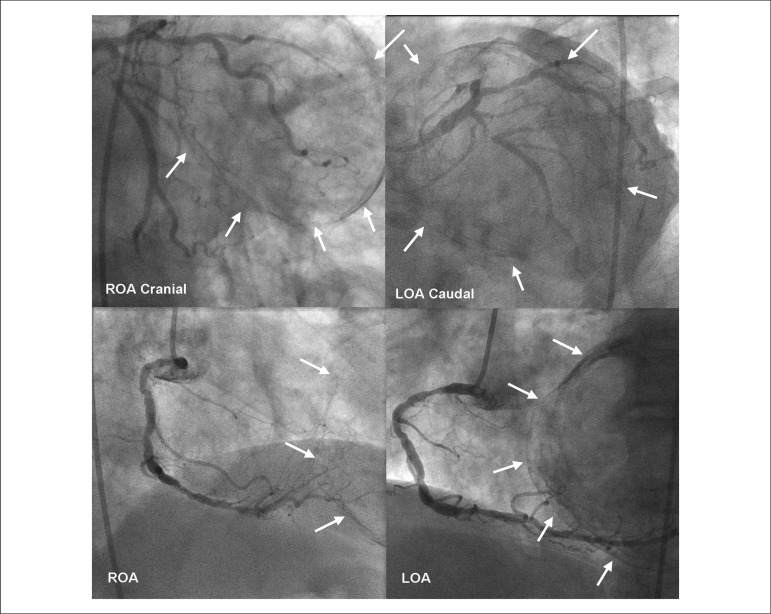
Coronary angiography showing severe multivessel disease. The white arrows outline the giant and calcified aneurysm. ROA: Right oblique anterior; LOA: Left oblique anterior.

**Figure 2:**
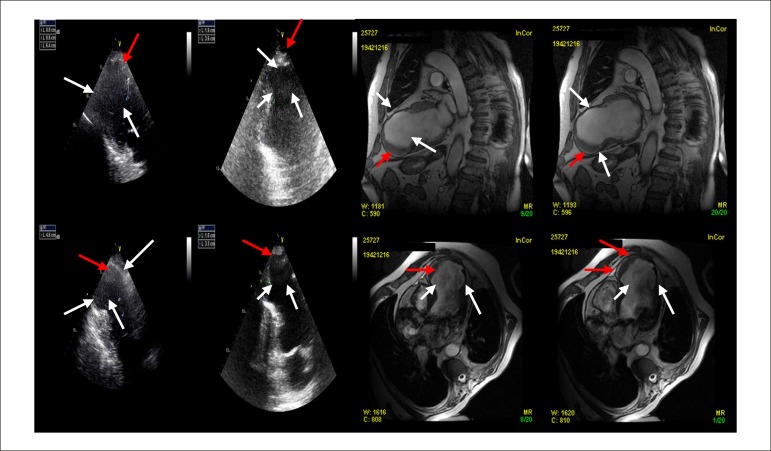
Left panel: Transthoracic echocardiogram showing dilated left chambers with a large apical thrombus (red arrows) and a giant antero-apical LV aneurysm (white arrows). Right panel: MRI findings of the reported aneurysm (white arrows) with a large apical thrombus (red arrows). LV, left ventricle; MRI, Magnetic Ressonance Imaging.

A 70-year-old male patient with a history of smoking, anterior MI, chronic obstructive pulmonary disease, chronic kidney disease, congestive heart failure (HF), and LV thrombus anticoagulated with warfarin was hospitalized for invasive stratification after an episode of high-risk unstable angina. Coronary angiography (Figure 1) revealed severe multivessel disease: right coronary artery (RCA) with multiple lesions, the larger one in its proximal segment; proximal occlusion of the left anterior descending artery (LAD); and proximal subocclusion of the left circumflex artery (LCx). The left ventriculography (Videos 1 and 2) evidenced a giant and calcified true LV aneurysm. LCx was considered the culprit vessel and subjected to an unsuccessful attempt of percutaneous coronary intervention (PCI). This PCI was complicated by a type II coronary perforation, solved by prolonged local balloon inflation and reversal of anticoagulation. The patient developed cardiogenic shock, received circulatory support by intra-aortic balloon pumping and intensive medical care with vasopressors (norepinephire and vasopressin), an inotrope (dobutamine), and invasive ventilatory assistance. Transthoracic echocardiogram (Figure 2, left panel) showed dilated left chambers (62 × 50 mm), poor LV ejection fraction (20% using Simpson's rule), a giant antero-apical LVA with a large apical thrombus (19 × 36 mm), and akinesia of the middle segments of the anterior, septal, and inferior LV walls. Magnetic resonance imaging (MRI) showed the absence of viability and transmural delayed enhancement in the anterior, anteroseptal, medium, and lower inferoseptal wall and all apical LV wall segments. There was a giant anteroapical LVA (65 × 59 × 6 5 mm; volume of 117 mL and indexed volume of 61 mL/m^2^) (Figure 2, right panel). After recovery from the critical status described above, the medical treatment was gradually optimized (aspirin 100 mg/day, clopidogrel 75 mg/day, atorvastatin 40 mg/day, metoprolol succinate 100 mg/day, enalapril 40 mg/day, spironolactone 25 mg/day, furosemide 80 mg/day, and warfarin). The patient had no impairment of his functional status, no complex ventricular arrhythmia, and no thromboembolic arterial events. He remained treated with a conservative strategy. Twenty months after hospital discharge, under the above described medical treatment for HF plus oral anticoagulation (warfarin), there were no readmissions because of cardiovascular reasons. In addition, there was no documentation of any thromboembolic event or malignant ventricular arrhythmia.

**Video 1:** Left ventriculography in right anterior oblique view.

**Video 2:** Left ventriculography in left anterior oblique view.

## Discussion

A true LVA usually involves the anterior wall. LVA may be asymptomatic; however, it can be the cause of refractory heart failure, sustained ventricular arrhythmias, and arterial embolism. Although the exact definition of LVA remains controversial, it is usually defined as a well-delineated, thin, scarred, or fibrotic wall, devoid of muscle or containing necrotic muscle as a result of a healed transmural MI. The involved wall segment is either akinetic or dyskinetic during systole. It was previously estimated that LVA develops in up to 30%-35% of patients with Q wave MI. However, its incidence is clearly decreasing, and it currently occurs in approximately 8%-15% of patients.^3^ This is because of the introduction of major improvements in the management of MI, such as thrombolytic agents, PCI, and the administration of afterload-reducing agents. As in our case, most LVAs are located in the anterior and/or apical walls a result of total occlusion of LAD and the absence of collateralization. Only 10%-15% involve the inferior wall because of right coronary artery occlusion. Lateral LVA secondary to LCx occlusion is exceedingly rare.

Echocardiography can usually distinguish a pseudoaneurysm (PA) from a true LVA by the appearance of the connection between the aneurysm and ventricular cavity. PA has a narrow neck, typically less than 40% of the maximal aneurysm diameter; this causes an abrupt interruption in the ventricular wall contour and turbulent flow by pulsed Doppler at the neck or within the cavity itself.^5,6^ In contrast, true LVAs are nearly as wide at the neck as at the apex.

One of the most reliable methods for the diagnosis of PA is coronary angiography and ventriculography, demonstrating a narrow orifice that leads to a saccular aneurysm and the lack of surrounding coronary arteries.

MRI can clearly localize the site of the aneurysm. An additional advantage includes the capability to distinguish between the pericardium, thrombus, and myocardium, which are not easily distinguished by ventriculography.^7^ Myocardial viability by MRI uses a delayed contrast-enhanced imaging technique for accurately delineating the infarct size and its extent. In the case of a true aneurysm, the tissue forming the wall of the aneurysm will show delayed enhancement, indicating the scar tissue as a result of the infarcted myocardium.^7^

The indications for left calcified ventricular aneurysmectomy remain controversial. Some authors advocate that the existence of recurrent ventricular arrhythmias, systemic embolization, and refractory congestive HF are good reasons for surgery. Previous studies have reported that surgical repair achieves worse outcomes than medical treatment in cases of satisfactory response to this approach. The aneurysmectomy associated with coronary artery bypass grafting (CABG) is reserved for refractory cases (a Class IIa recommendation according to the American College of Cardiology/American Heart Association guidelines), with no impact on functional class improvement, reduction in mortality, or hospitalization rates from cardiovascular disease according to the STICH trial.^8,9^

During the course of hospitalization, our patient did not experience any new impairment of his functional status, neither with complex arrhythmias nor with thromboembolic arterial events. Therefore, we proposed a conservative treatment strategy.
